# Supplementary material for: Sustainable production of highly conductive multilayer graphene ink for wireless connectivity and IoT applications
Source: Nat Commun. 2018 Dec 5;9:5197. doi: 10.1038/s41467-018-07632-w (PMC6281590; doi:10.1038/s41467-018-07632-w)
Supplement: Supplementary file 1 — Supplementary Information [file 41467_2018_7632_MOESM1_ESM.pdf]

Supplementary Information

Sustainable Production of Highly Conductive Multilayer Graphene Ink for Wireless Connectivity and IoT Applications

Authors: Kewen Pan[1], Yangyang Fan[2], Ting Leng[1], Jiashen Li[2], Zhiying Xin[2], Jiawei Zhang[1], Ling Hao[4], John Gallop[4], Kostya S. Novoselov [3,5] and Zhirun Hu[1,5]*.

[1]School of Electrical and Electronic Engineering, University of Manchester, Manchester, M13 9PL, United Kingdom.

[2]School of Materials, University of Manchester, Oxford Rd, Manchester, M13 9PL, United Kingdom.

[3]School of Physics and Astronomy, University of Manchester, Manchester, M13 9PL, United Kingdom.

[4]National Physical Laboratory, Hampton Road, Teddington, TW11 0LW, United Kingdom.

[5]National Institute of Graphene, Manchester, M13 9PL, United Kingdom.

[*]Correspondence and requests should be addressed to Zhirun Hu (email: z.hu@manchester.ac.uk)

Supplementary Figures:

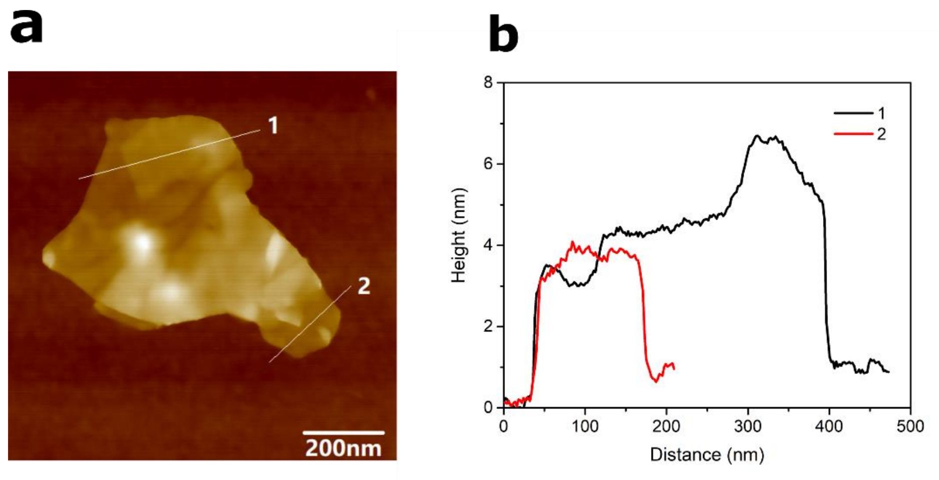

Supplementary Figure 1: A single graphene nanoflake profile measurement. Atomic force microscopy image of a single graphene nanoflake exfoliated in Cyrene **a** and the corresponded cross section heights **b**.

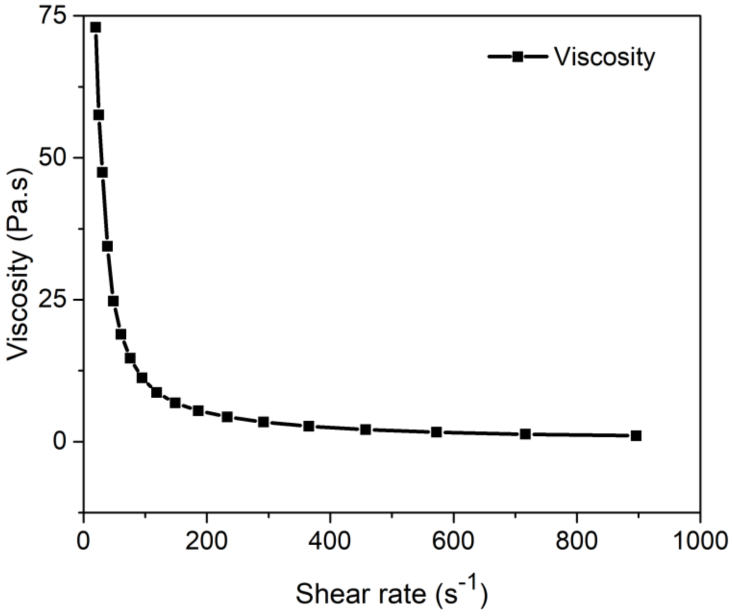

Supplementary Figure 2: Viscosity of the screen printable ink. Measured viscosity data of the screen printable ink with the graphene concentration of 70 mg mL^{-1} and CAB concentration of 7 mg mL^{-1}.

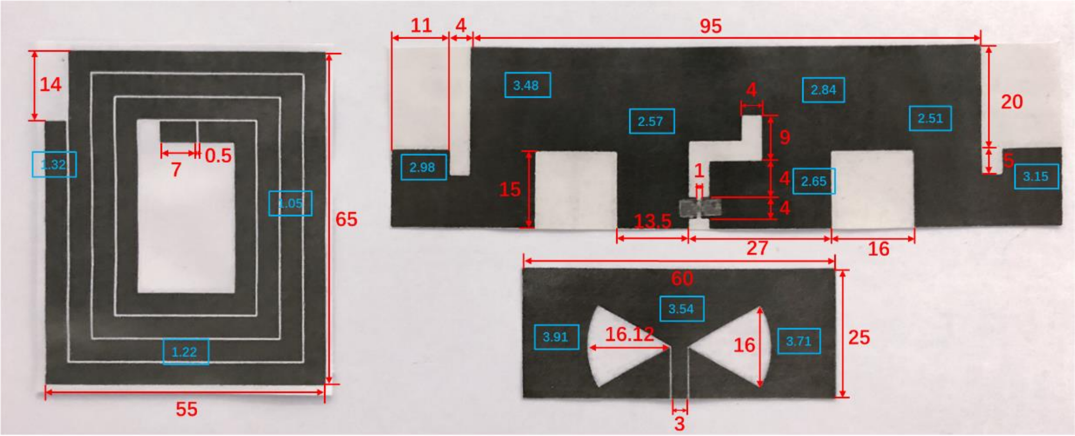

Supplementary Figure 3: Antenna dimensions and sheet resistance. The blue rectangles represent measured sheet resistance at this point. The variation was caused by unflatness of the exposed screen. The average sheet resistance for the nearfield antenna is 1.2 Ω sq^{-1}; for RFID antenna is 2.88 Ω sq^{-1}; for wideband slot antenna is 3.72 Ω sq^{-1}. The red lines indicate specific dimensions (mm).

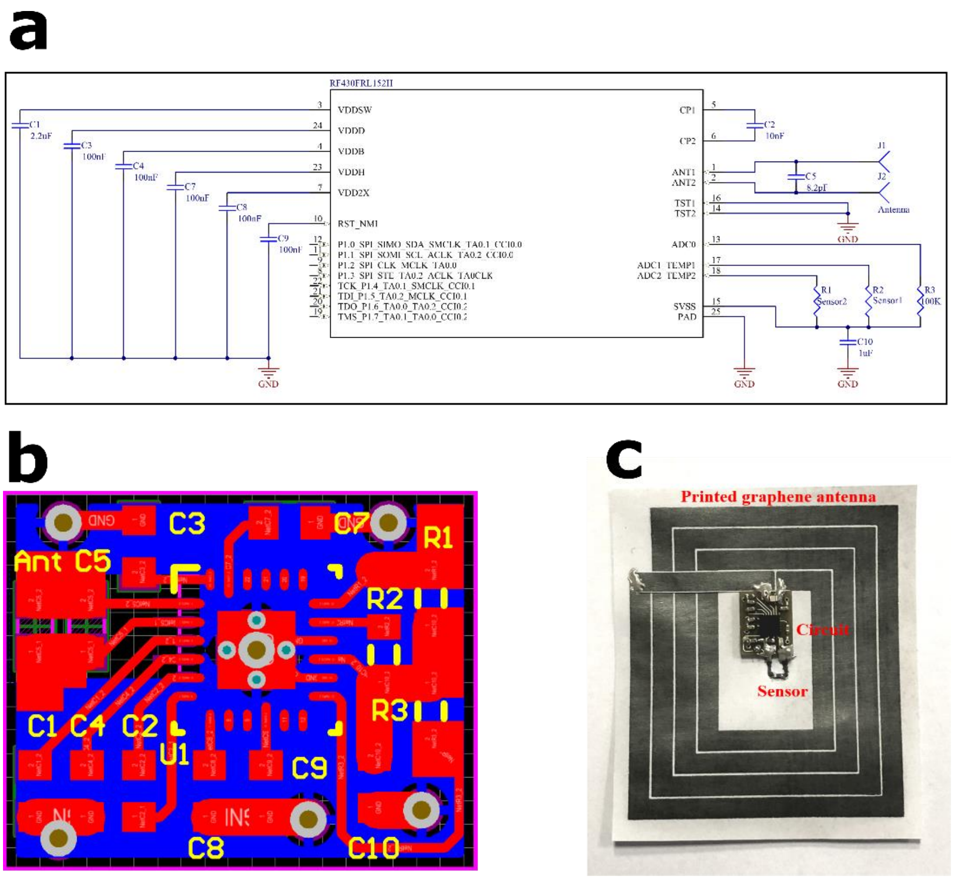

Supplementary Figure 4: Printed graphene temperature monitoring system. Schematic sensing and conversion circuit **a** and PCB **b**. Photo of the entire wireless sensor **c**. The conductive epoxy (CW2400, Circuitworks) was used to connect graphene antenna and the PCB.

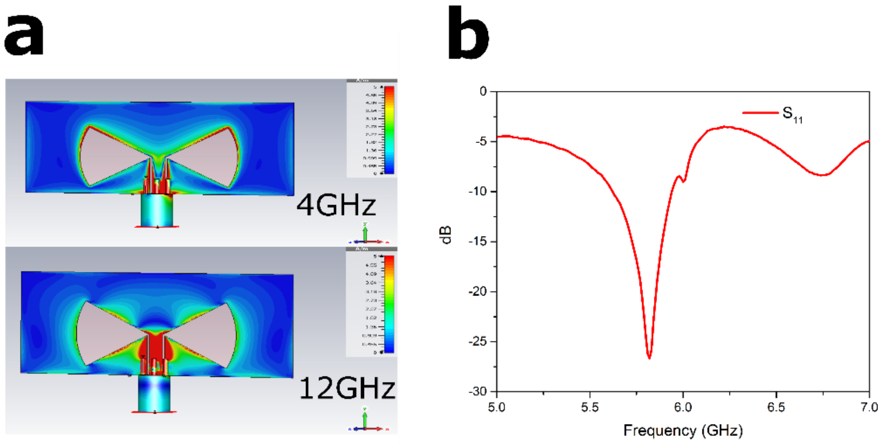

Supplementary Figure 5: Energy harvesting Antenna and circuits. Surface current distributions of the C-X-Ku band wideband antenna at fundamental resonance (4GHz) and harmonic resonance (12GHz) **a**. Reflection coefficient of matched conversion circuit **b**.

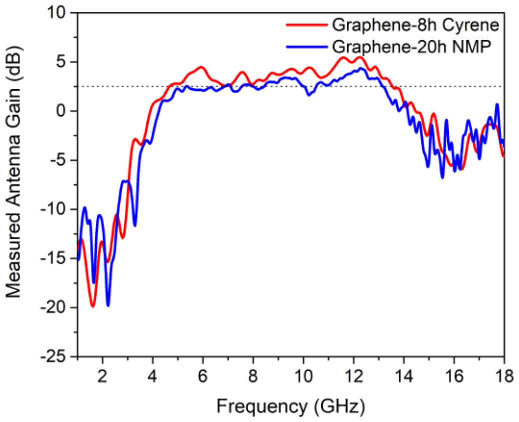

Supplementary Figure 6: Antenna gain based on different graphene inks. Red line: Cyrene based graphene ink (8h ultrasonic treatment). Blue line: NMP based graphene ink (20h ultrasonic treatment). The 8h Cyrene based antenna has slightly higher gain than 20h NMP based antenna because the conductivity of 8h Cyrene printed pattern is slightly higher.

Supplementary Notes:

Supplementary Note 1: Temperature monitoring system converting circuits.

In order to demonstrate the functionality of the graphene printed NFC antenna, a commercial NFC chip (RF430FRL152H) which is integrated with NFC module and microcontroller was connected to the antenna. The capacitor C5 in Supplementary Figure 3a together with internal capacitor of the NFC chip constitute an LC resonance circuit which operates in 13.56 MHz. The RTD temperature sensor (NTHS0603N17N2003JE, VISHAY) in Supplementary Figure 3c was connected to R1 and R3, which are standard resistors. The absolute measurement accuracy is 0.3% when refresh frequency is 0.5 Hz.
